# Hormonal regulation of alveolarization: structure-function correlation

**DOI:** 10.1186/1465-9921-7-47

**Published:** 2006-03-27

**Authors:** Samuel J Garber, Huayan Zhang, Joseph P Foley, Hengjiang Zhao, Stephan J Butler, Rodolfo I Godinez, Marye H Godinez, Andrew J Gow, Rashmin C Savani

**Affiliations:** 1 Division of Neonatology, Department of Pediatrics, Children's Hospital of Philadelphia, University of Pennsylvania School of Medicine, Philadelphia, PA, USA; 2 Department of Anesthesiology and Critical Care Medicine, Children's Hospital of Philadelphia, University of Pennsylvania School of Medicine, Philadelphia, PA, USA; 3 Division of Neonatal-Perinatal Medicine, Division of Pulmonary and Vascular Biology, Room K4.224, University of Texas Southwestern at Dallas, Dallas, TX USA

## Abstract

**Background:**

Dexamethasone (Dex) limits and all-trans-retinoic acid (RA) promotes alveolarization. While structural changes resulting from such hormonal exposures are known, their functional consequences are unclear.

**Methods:**

Neonatal rats were treated with Dex and/or RA during the first two weeks of life or were given RA after previous exposure to Dex. Morphology was assessed by light microscopy and radial alveolar counts. Function was evaluated by plethysmography at d13, pressure volume curves at d30, and exercise swim testing and arterial blood gases at both d15 and d30.

**Results:**

Dex-treated animals had simplified lung architecture without secondary septation. Animals given RA alone had smaller, more numerous alveoli. Concomitant treatment with Dex + RA prevented the Dex-induced changes in septation. While the results of exposure to Dex + RA were sustained, the effects of RA alone were reversed two weeks after treatment was stopped. At d13, Dex-treated animals had increased lung volume, respiratory rate, tidal volume, and minute ventilation. On d15, both RA- and Dex-treated animals had hypercarbia and low arterial pH. By d30, the RA-treated animals resolved this respiratory acidosis, but Dex-treated animals continued to demonstrate blood gas and lung volume abnormalities. Concomitant RA treatment improved respiratory acidosis, but failed to normalize Dex-induced changes in pulmonary function and lung volumes. No differences in exercise tolerance were noted at either d15 or d30. RA treatment after the period of alveolarization also corrected the effects of earlier Dex exposure, but the structural changes due to RA alone were again lost two weeks after treatment.

**Conclusion:**

We conclude that both RA- and corticosteroid-treatments are associated with respiratory acidosis at d15. While RA alone-induced changes in structure andrespiratory function are reversed, Dex-treated animals continue to demonstrate increased respiratory rate, minute ventilation, tidal and total lung volumes at d30. Concomitant treatment with Dex + RA prevents decreased septation induced by Dex alone and results in correction of hypercarbia. However, these animals continue to have abnormal pulmonary function and lung volumes. Increased septation as a result of RA treatment alone is reversed upon discontinuation of treatment. These data suggest that Dex + RA treatment results in improved gas exchange likely secondary to normalized septation.

## Background

Bronchopulmonary Dysplasia (BPD) remains a significant cause of morbidity and mortality affecting as many as 30–40% of infants born less than 30 weeks gestation [1]. While the pathophysiology of BPD includes both inflammatory and fibrotic processes, a critical component is an arrest of lung development at the saccular stage and failure of alveolarization [1]. Alveolar hypoplasia has been documented in preterm humans [2] as well as in preterm baboons born at 75% gestation and ventilated for the first two to three weeks of life [3].

Lung development is a dynamic process consisting of embryonic, pseudoglandular, canalicular, saccular, and alveolar stages marked by the progression from a rudimentary lung bud to a saccule with a completely developed respiratory tree. In the human, alveolarization begins during the 36^th ^week of gestation and continues for at least 3 years after birth [4]. The development of alveoli involves the formation of crests, or secondary septae, at precise sites of the saccular wall. These crests protrude into the saccular air space, include the inner layer of the capillary bilayer, and further subdivide the saccule into subsaccules that later become mature alveoli. While not fully understood, the regulation of this process involves several cell types including endothelial cells, myofibroblasts, and epithelial cells as well as growth factors, hormones, and environmental conditions that either inhibit or stimulate alveolar growth [5].

The stages of lung development are the same in rodents except that alveolar formation is an entirely postnatal event occurring in the first three weeks of life [6,7]. Interestingly, in rodents, alveolarization is associated with decreased plasma corticosteroid concentrations [8], and administration of corticosteroids during this period inhibits alveolarization [9]. Using a neonatal rat model, Massaro and others have demonstrated the effects of Dexamethasone (Dex) and all-trans-retinoic acid (RA) treatment on alveolar development [10]. Dex-treated animals develop a simplified architecture with large terminal sacs, whereas RA-treated animals develop smaller, more numerous alveoli. Dex-induced changes are prevented in animals that receive either concomitant Dex + RA administration [10] or RA after earlier treatment with Dex alone, even though RA is given after the period known to be critical for alveolar development [11].

While considerable information is available for hormonally mediated structural changes during alveolarization, there is a paucity of information on the impact of such hormonal manipulations and the resultant architectural alterations on pulmonary function. Srinivasan et al. [12] measured pulmonary function in rats treated with Dex and/or RA in the first two weeks of life. In their studies, changes in lung volume and compliance resulting from Dex treatment alone were not reversed with simultaneous RA administration [12]. However, since Srinivasan's study was done in sedated 30–39 day old animals, it is unclear if functional effects of altered alveolarization are evident in normally breathing rats. In addition, no information on arterial oxygen or carbon dioxide homeostasis or exercise tolerance was currently available for this model.

The goal of the current study was to determine the structure-function relationships after glucocorticoid and retinoid treatment in neonatal rat pups undergoing alveolarization. We report that both RA and Dex-induced alteration of alveolarization was associated with hypercarbia at two weeks. However, only Dex-treated animals had larger lung volumes with increased respiratory rate and tidal volume. Concomitant RA treatment prevented the Dex-induced changes in secondary septation and corrected the respiratory acidosis. However, Dex + RA-treated animals continued to have increased respiratory rate, tidal volume, minute ventilation, and larger lung volumes. Treatment with RA alone increased the number of alveoli as measured by radial alveolar counts, but this response was reversed two weeks after stopping treatment, even if the RA treatment was given later, after the optimal time for alveolarization.

## Methods

### Animals

All protocols were reviewed and approved by the Children's Hospital of Philadelphia (CHOP) Institutional Animal Care and Use Committee in accordance with NIH guidelines. Timed pregnant Sprague-Dawley rats (Charles River Breeding Laboratory, Wilmington, MA), were maintained until parturition on a 12:12 h light:dark cycle with unlimited access to food (Purina Lab Diet, St. Louis, MO) and water in the Laboratory Animal Facility at CHOP.

### Day 15/30 protocol

After birth, litters were adjusted to 10 pups per litter within 12 h of birth and divided into the following treatment groups: (1) Dexamethasone (Dex, American Regent Laboratories, Inc., Shirley, NY) 0.1 μg in 20 μl 0.9%NaCI [saline]) or saline alone (20 μl) subcutaneously (SQ) daily from days 1–14; (2) all-trans-retinoic acid (RA, Sigma-Aldrich, St. Louis, MO) 500 μg/kg in 20 μl cottonseed oil (CSO, Sigma-Aldrich, St. Louis, MO) or CSO alone (20 μl) via intraperitoneal (IP) injection daily days 3–14; (3) Dex and RA at doses and days as above; (4) saline and CSO at doses and days above; and (5) control (no injections). The dose of Dex was based on previous literature demonstrating only mild effects on somatic growth [9]. Because it was difficult to discern the gender of rats at birth, both males and females were studied at days 1,5, 10, 15, and 30 as described below.

### Extended (Day 37/52) study protocol

This study was designed to evaluate the structural consequences of RA administered after the critical period for alveolar development in rats previously treated with Dex from days 1 to 14. Pups were normalized to 10 per litter shortly after birth and, in addition to a control group (no injections), were divided into the following groups receiving either saline or Dex SQ daily on days 1–14 followed by either IP CSO or RA daily on days 24–36: (1) Early Saline + Later CSO; (2) Early Saline + Later RA; (3) Early Dex + Later CSO; (4) Later Dex + Later RA. Animals were weaned from their mothers at d21 and divided by sex into separate cages (n = 2–3 per cage). The doses of saline, Dex, and CSO were as above. Males and females were studied independently as described below on d37 (after 2 weeks of RA treatment) and d52 (2 weeks after stopping RA treatment).

### Lung harvest

Anesthesia for all studies was attained using an intramuscular injection of a Ketamine/Xylazine (87:13 μg/kg) cocktail. The right lung was removed, snap frozen in liquid nitrogen, and stored at -80°C for future analysis. As has been previously described [13], the left lung was inflated to 25 cm H_2_O pressure with formalin and stored in formalin for 24 hours. Lungs were then processed to obtain 5-micron thick paraffin sections.

## Structural analyses

### Histology

For each time point, sections were stained with hematoxylin and eosin in order to examine lung architectural differences using light microscopy. Both 40x and 100x images were obtained using a Nikon TE 300 inverted microscope.

### Radial alveolar counts (RAC)

To quantify alveolarization, RAC were obtained as described by Emery and Mithal [14] and validated by Cooney and Thurlbeck [15]. These investigators confirmed that forty measurements per lung were sufficient to establish a reliable morphometric assessment of alveolarization. Briefly, a perpendicular line was drawn from the last respiratory bronchiole to either the pleura or the nearest connective tissue septum. Using low power images, over 90% of all lines drawn were to the pleura. A minimum of forty lines for each lung were drawn and the number of septae intersected were counted for each line. In addition, at least three sections from several levels within the tissue block were used for each animal.

## Functional analyses

### Plethysmography

On d13, pups were placed in a dual chamber plethysmograph (Buxco Electronics Inc, Sharon, CT) for non-invasive, non-sedated, real-time measurement of pulmonary function. This airtight system, which separates the head from the body by a latex collar barrier, measures airflow across a pneumotach plate and uses a flow transducer to determine various parameters including respiratory rate (RR), tidal volume (TV), and minute ventilation (MV). Animals were acclimated to the chamber until consistently normal breathing patterns were noted. Thermal neutrality was maintained throughout the study period for each animal. Measurements were made twice, each for two minutes with only data that were consistently within 5% variance of each other used for analysis. TV and MV were normalized to body weight. We were unable to obtain measurements at d30 as the rats were too large for the dual chamber plethysmograph.

### Arterial blood gases (ABG)

To evaluate the efficiency of gas exchange, an ABG was obtained from the distal aorta at the time of harvest for d15 and d30 animals. While animals were spontaneously breathing under adequate anesthesia, the abdomen was opened. With the diaphragm left intact, the distal aorta was identified, and a sample drawn using a heparinized syringe. The harvest then proceeded as described above. Samples were analyzed using an i-STAT Portable Clinical Analyzer (i-STAT Corporation, East Windsor, NJ).

### Lung volume of displacement

At d15, lungs were inflated to a pressure of 25 cm H2O with 10% formalin, harvested en bloc and fixed overnight. Lung volume was measured by waterdisplacement immediately after inflation with maintenance of inflation confirmed by repeat measurement 24 hours after fixation.

### Pressure-Volume (PV) studies

Separate animals were studied at d30 to obtain PV curves. After appropriate anesthesia, the trachea was cannulated and the animals were placed on a Harvard rodent ventilator (Harvard Apparatus Inc., Holliston, MA). Animals were ventilated with 100% O_2 _for 10 minutes after which time the cannula was sealed by closing the stopcock to allow the lungs to degas. PV curves were obtained with the chest closed. Inflation and deflation of the lungs was performed in 0.5 ml air increments and pressure was measured by an HP Omni Care (Wolfpham, MA) using an Abbott pressure transducer (HP M1006B pressure modulator, North Chicago, IL). Maximum inflation was achieved at 33 mmHg (25 cm H_2_O) and maximum deflation was achieved by the corresponding withdrawal of air to decrease pressure to 0 mmHg. Only lungs that did not leak were included for analysis.

### Analysis of PV curves

Regression analysis using Sigma Plot 8.0 (Systat Software Inc., Port Richmond, CA) generated best-fit models for inflation and deflation curves using data for all animals in each treatment group. For inflation, a sigmoidal 3 parameter model was utilized [] where "y" is the lung volume, "a" is an estimate of the maximum lung volume (Vmax), "x" is the pressure at a given volume, "x_0_" is the pressure at a volume of 0, and "b" is a constant. The deflation model was based on an exponential rise model [y = a(1-e^-bx^)]. The parameters within this model provided an estimate of Vmax and the standard error of the estimate. First derivative curves were used to determine maximum compliance and the pressure at which this was achieved, while second derivative curves were used to calculate points of maximum acceleration and deceleration during inflation and deflation. Hysteresis was defined as the area bound by the inflation and deflation curves. To quantify differences between treatment groups, the area was obtained and averaged for each treatment group. All parameters were adjusted to body weight in kilograms.

### Forced exercise swim testing

Separate groups of animals underwent forced swim testing to evaluate exercise tolerance at d15 and d30. Rats were placed in a tank filled with water at 24°C at a level high enough to prevent their tails from touching the bottom of the tank. They remained in the water until the first sign of fatigue manifested by their entire body sinking below the water level. They were then rescued and harvested a day later as described above.

### Statistical analysis

For all variables measured, values were expressed as mean ± SE, using the number of animals rather than the number of observations for calculations. ANOVA and two-tailed t-tests assuming unequal variances with Tukey correction were used to determine intergroup significance with a p-value <0.05 considered statistically significant for all analyses.

To analyze PV curves, z-scores were used for comparison of Vmax between treatment groups with a score >1.96 considered significant at p < 0.05. For analysis of maximum compliance and pressure at which it was achieved, rate of maximum deflation, and hysteresis, an ANOVA and unpaired t-test with Tukey correction were used with p < 0.05 considered significant for all analyses.

## Results

### Day 15/30 protocol

Neonatal rats were exposed to either saline or Dex and/or CSO or RA for the first two weeks of life as described in Methods. We first sought to reproduce the structural alterations from hormonal treatments during the critical period of alveolar development in rats [10]. Animals were examined at postnatal days 1, 5, 10, 15, and 30.

### Weight gain

All animals were the same weight at the start of the experiment (6.7 ± 0.1 grams, n = 60), and litter sizes were normalized to 10 per litter to ensure equal access to nutrition. Table 1 shows the weights of rats given various treatments throughout the first two weeks and at d30 of life. Dex- and Dex + RA-treated pups had significantly less weight gain compared to saline- or Saline + CSO-treated animals by day 5 (Table 1). At d 15, Dex and Dex + RA pups weighed approximately 15% less than corresponding controls. Rats treated with RA alone had weight gain comparable to control animals at all time points. Despite stopping hormonal treatments at d14, the body weights of Dex- and Dex + RA-treated animals continued to be significantly lower (about 20%) than controls at d30 (Table 1).

**Table 1:** Body weights in grams: Though no differences in body weight were noted at birth between groups (6.7 ± 0.1 grams, n = 60), the effect of Dex on weight gain was evident by day 5 and continued until d30 as both Dex and Dex + RA pups had significantly lower weights compared to saline controls. RA treatment alone did not affect weight. Values are expressed mean ± SE. *p < 0.05 vs. corresponding controls.

**Treatment Group**	**Day 5**	**Day 10**	**Day 15**	**Day 30**
Saline/Saline + CSO (n = 8–15)	12.9 ± 0.4	17.1 ± 0.4	31.4 ± 0.8	164 ± 7
Cottonseed oil (CSO) (n = 8–15)	13.2 ± 0.3	16.6 ± 0.6	29.1 ± 1.0	139 ± 13
Retinoic Acid (RA) (n = 8–15)	12.9 ± 0.3	16.8 ± 0.4	28.8 ± 0.8	150 ± 8
Dexamethasone (Dex) (n = 8–16)	11.4 ± 0.2*	15.0 ± 0.2*	25.8 ± 0.7*	126 ± 6*
Dex + RA(n = 7–14)	11.7 ± 0.2*	15.4 ± 0.3*	26.0 ± 0.9*	125 ± 10*

### Morphology

**Figure 1:**
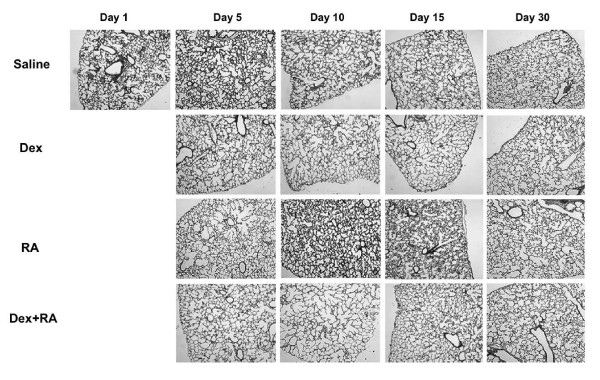
Changes in morphology during hormonal treatments at days 1, 5, 10, 15, and 30: Dex-induced changes in architecture were evident as early as d5 and persisted to d30. RA-induced changes were also evident at d5, continued to d15, but had reversed at d30. Concomitant Dex and RA administration resulted in septation similar to that of controls between d10 and d15 with continued normal appearance at d30. Dex: Dexamethasone. RA: all-*trans*-retinoic acid, (all images 40× magnification)

**Figure 2:**
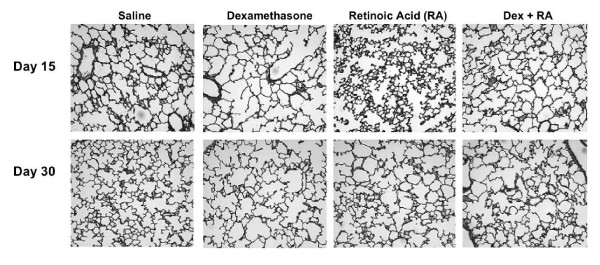
Days 15 (top) and 30 (bottom) histology: A simplified distal architecture was seen in Dex-treated animals at both days. At d15, RA-treated pups had smaller more numerous alveoli, but these changes were no longer seen at d30. Dex + RA treatment resulted in a restitution of septation to near that of saline controls at both days, (all images 100× magnification)

Hormonal treatment of rat pups during the period of alveolar development resulted in alterations of lung architecture. At d15, Dex-treated animals appeared to have larger, simpler distal air spaces than saline controls. These structural changes were evident as early as d5 (Figures 1 and 2) and, despite discontinuation of treatment at d14, persisted to d30. RA-treated pups, on the other hand, appeared to have smaller, more numerous alveoli than CSO controls as early as d5 and up to d15. Interestingly, rats treated with RA alone up to 14 days and examined at d30 had lung histology similar to that of control animals (Figure 2), demonstrating a loss of the RA effect within two weeks of stopping treatment. Meanwhile, Dex + RA-exposed pups showed a simplified distal architecture similar to Dex alone pups at days 5 and 10. The corticosteroid-induced changes in architecture were prevented by days 10 to 15 with concomitant RA treatment such that, at d15, they displayed a distal lung structure similar to that of controls. In contrast to animals treated with RA alone, the effect of concomitant Dex + RA treatment was sustained to d30 (Figure 2).

### Radial alveolar counts (RAC)

**Figure 3:**
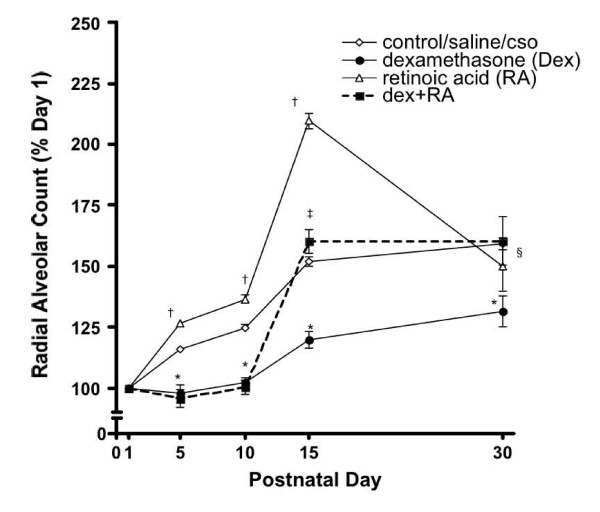
Radial alveolar counts (RAC) as a percentage of day 1: Changes in RAC were seen as early as day 5 with RA alone-treated animals having significantly higher counts at days 5, 10, and 15. However, at d30, RA-treated animals had counts similar to controls. Dex alone-treated animals had significantly lower RAC at each time point studied. (*p < 0.05 vs. saline-treated animals; ^†^p < 0.05 vs. CSO-treated animals, ^‡^p = 0.49 vs. saline-treated animals, ^§^p = 0.58 vs. CSO-treated animals) CSO: cottonseed oil.

Morphometric evaluation of alveolarization was achieved using RAC. (Figure 3). Compared to controls, and in accordance with histological appearance, RAC were significantly lower in Dex-treated and significantly higher in RA-treated pups at days 5, 10, and 15. Dex + RA animals had lower RAC compared to saline controls at days 5 and 10, but by day 15, rats treated with both hormones had RAC that were similar to controls (Figure 3). At d30, while RAC remained significantly lower in Dex-treated animals compared to saline controls, the RAC for both RA- and Dex + RA-treated animals were similar to controls (Table 2).

**Table 2:** Radial alveolar counts (RAC) at d15 and d30: RAC at d15 were significantly lower in Dex-treated and higher in RA-treated pups while Dex + RA animals were similar to controls. At d30, RAC continued to be significantly lower in Dex-treated pups but RA alone increases were lost demonstrating reversal upon stopping treatment. Values are expressed mean ± SE. *p < 0.05 vs. saline-treated animals. ^†^p < 0.01 vs. CSO-treated animals. ^‡^p < 0.01 vs. d15 RA animals.

**Treatment Group**	**Day 15**	**Day 30**
Control (n = 3–4)	8.5 ± 0.2	9.0 ± 0.3
Saline (n = 3–4)	8.7 ± 0.3	8.8 ± 0.3
Cottonseed oil (CSO) (n = 3–4)	8.5 ± 0.2	8.8 ± 0.1
Retinoic Acid (RA) (n = 3–4)	11.6 ± 0.2^†^	8.9 ± 0.1^‡^
Dexamethasone (Dex) (n = 3–4)	6.6 ± 0.2*	7.3 ± 0.4*
Dex + RA (n = 3–4)	8.9 ± 0.3	8.5 ± 0.2

### Plethysmography

In order to determine the functional consequences of hormonally altered lung architecture, a number of variables of pulmonary function were examined at d13 (Table 3). In association with decreased RAC, Dex-treated animals had a significantly increased RR, TV, and MV compared to saline controls. While concomitant treatment with RA (Dex + RA) reversed RAC as compared to Dex alone, this treatment had no effect on the increased RR, MV, or TV seen in association with Dex alone. RA alone, a treatment that increased RAC, had no effect on RR, MV, or TV (Table 3).

**Table 3:** Plethysmography at d13: Dex-treated animals showed an increased respiratory rate (RR), tidal volume, and minute ventilation compared to saline controls. Retinoic acid treatment alone did not alter RR but, when given with Dex, resulted in decreased RR similar to that of controls. Values are expressed mean ± SE. *p < 0.05 vs. saline; ^†^p = 0.3 vs. Dex; ^‡^p = 0.5 vs. Dex; ^§^p = 0.9 vs. Dex. bpm: breaths per minute

**Treatment Group**	**Respiratory Rate (bpm)**	**Tidal Volume (ml/kg)**	**Minute Ventilation (ml/kg)**
Control (n = 8)	171 ± 20	5.2 ± 0.9	861 ± 145
Saline (n = 9)	180 ± 5.0	4.8 ± 0.8	890 ± 163
Cottonseed oil (CSO) (n = 14)	179 ± 12	6.5 ± 0.7	935 ± 109
Retinoic acid (RA) (n = 15)	180 ± 6.0	5.2 ± 0.7	810 ± 103
Dexamethasone (Dex) (n = 16)	211 ± 11*	8.1 ± 0.8*	2260 ± 150*
Dex + RA (n = 14)	195 ± 9.0^†^	8.7 ± 1.0^‡^	2131 ± 186^§^

### ABG

Since significantly increased RR, MV, and TV were observed on d13 in Dex- and Dex + RA-treated animals, we examined ABGs to evaluate gas exchange both at d15 and d30 (Table 4). On d15, when RA alone and Dex alone treatment altered distal lung architecture, both sets of rat pups had hypercarbia with respiratory acidosis. On d30, when the RA alone-treated animals had RAC similar to those of controls, the RA-alone animals had normal pH and PCO_2_. Also at d30, Dex alone-treated animals, that had persistentlylarger distal air spaces, continued to have hypercarbia with a respiratory acidosis. However, Dex + RA-treated animals at both d15 and d30 had pCO_2 _values no different from control despite continued increased RR, MV, and TV. Interestingly, oxygenation was lower in the d15 group given RA alone. Dex + RA animals at d15, however, had PO_2 _values no different from those of controls (Table 4). These data suggest impaired gas exchange in Dex alone-treated animals with a failure of secondary septation, and, despite continued tachypnea and increased minute ventilation, a correction of this abnormality occurred with concomitant RA treatment.

**Table 4:** Arterial blood gases at d15 and d30: Dex- or RA-treated animals at d15 had a respiratory acidosis with hypercarbia (*p < 0.01 vs. saline/controls) and this was maintained in Dex-treated animals at d30 (*p < 0.01 vs. saline/controls). Day 15 animals given Dex + RA did not have respiratory acidosis compared to Dex alone pups (^†^p < 0.05 vs. Dex alone). Animals at d30 that had been given Dex + RA showed a correction of pH and pCO2 (^†^p < 0.05 vs. Dex). Only d15 RA-treated animals had significantly lower pO2 values compared to controls (**p < 0.05 vs. saline/controls). Values are expressed mean ± SE.

**Treatment group**	**pH**	**pCO_**2**_**	**pO_**2**_**
**Day 15**			

Control/Saline (n = 6)	7.39 ± 0.03	41.9 ± 3.2	89.8 ± 6.4
Retinoic acid (RA) (n = 4)	7.29 ± 0.03*	54.2 ± 1.6*	67.3 ± 7.1**
Dexamethasone (Dex) (n = 7)	7.27 ± 0.01*	56.3 ± 3.4*	76.0 ± 9.4
Dex + RA (n = 4)	7.31 ± 0.04^†^	50.1 ± 6.0^†^	79.3 ± 2.9

**Day 30**			

Control/Saline (n = 12)	7.38 ± 0.01	47.7 ± 1.0	83.5 ± 2.8
Retinoic acid (RA) (n = 10)	7.38 ± 0.01	50.0 ± 1.5	80.1 ± 3.7
Dexamethasone (Dex) (n = 12)	7.33 ± 0.01*	55.5 ± 1.4*	80.9 ± 5.3
Dex + RA (n = 6)	7.38 ± 0.02^†^	48.5 ± 2.0^†^	83.7 ± 3.9

### Lung volume of displacement

As hypercarbia could result from increased dead space with larger lung volumes, we determined the lung volumes of displacement of hormonally treated animals at d15 of life (Table 5). Both Dex- and Dex + RA-treated pups had volumes of displacement that were significantly greater than those of control, saline or RA-treated animals, suggesting that Dex treatment is associated with larger lung volumes and that concomitant RA-treatment does not prevent this.

**Table 5:** Volumes of displacement on day 15: Lungs were inflated to 25 cm H_2_O, dissected en bloc and fixed overnight. The displacement of water by these lungs was determined and normalized to body weight in grams. Both Dex- and Dex + RA-treated animals had increased volumes of displacement as compared to controls and RA-treated animals (* p < 0.01).

**Treatment Group – d15**	**V_**disp**_/body weight (mL/g)**
Control/Saline (n = 9)	43.7 ± 1.28
Retinoic Acid (RA) (n = 7)	46.5 ± 1.28
Dexamethasone (Dex) (n = 6)	53.1 ± 3.13*
Dex + RA (n = 4)	55.5 ± 3.02*

### PV curves

**Figure 4:**
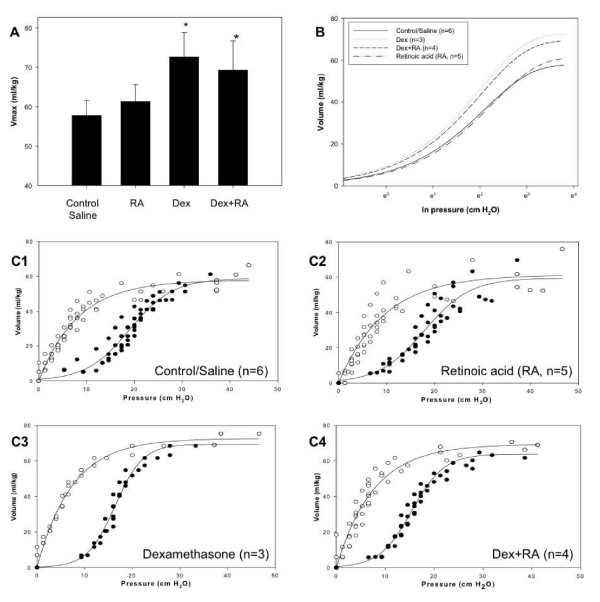
PV curves at d30: **(A1-4.) **Best-fit inflation and deflation curves for each treatment group: A significantly increased hysteresis was noted in the Dex group versus all other groups (p < 0.05). Data points represent individual measurements for each animal. **(B.) **Deflation curves for each treatment group generated from an exponential rise model. **(C.) **A significant increase in maximum volume (Vmax) existed between Dex vs. Control/Saline curves (*z = 2.05) as well as Dex + RA vs. Control/Saline (*z = 2.05). No difference was found between RA vs. Control/Saline (z = 0.6).

In order to confirm the lung volume of displacement measurements made at d15 and to evaluate lung volumes using an independent method, PV curves were generated on d30 as described in Methods. As shown in Figure 4A and 4C, Vmax was significantly increased in both Dex and Dex + RA compared to control/saline (z = 2.05). No difference existed between Dex compared to Dex + RA curves (z = 0.13) and Control/Saline versus RA curves (z = 0.6). The deflation curve for each treatment group was based on an exponential rise to maximum model (Figure 4B). The best-fit inflation and deflation curves generated for each treatment group are shown in Figure 4C1-4, with dots representing individual measurements for each animal. A significantly increased hysteresis was noted in Dex versus all other groups (Dex: 739 ± 19; Control/Saline: 566 ± 41; RA: 572 ± 66; Dex + RA: 594 ± 24 (ml/kg)^2^, p < 0.05, n = 3–6 per group). Collectively, these data suggest that Dex treatment resulted in larger lung volumes that concomitant RA treatment failed to abrogate.

The pressure required to reach the point of maximum compliance during inflation was lower in Dex vs. Control/Saline (16.3 ± 0.3 vs. 18.4 ± 0.6 mm H_2_O/kg, p = 0.014, n = 3–6 per group) and between RA and Dex + RA curves (17.9 ± 0.3 vs. 15.3 ± 0.3 mm H_2_O/kg, p < 0.01, n = 3–6 per group). In addition, the rate of maximal deflation was significantly greater in Dex vs. Control/Saline curves (9.6 ± 0.3 vs. 5.5 ± 0.4 mm H_2_O/kg/s, p < 0.05, n = 3–6 per group). The rate of maximal deflation tended to be lower in Dex vs. Dex + RA curves but this did not reach significance (9.6 ± 0.3 vs. 7.2 ± 0.9 mm H_2_O/kg/s, p = 0.07, n = 3–6 per group). This parameter was similar between RA and Dex + RA curves (7.4 ± 1.3 vs. 7.2 ± 0.9 mm H_2_O/kg/s, p = 0.88, n = 3–6 per group). These data suggest that Dex treatment resulted in lungs that had larger residual volumes requiring higher pressures to achieve the point of maximal compliance but were less stable during deflation.

Taken together, these physiologic data demonstrate that Dex + RA treatment failed to prevent larger lung volumes, RR, MV, and TV seen with Dex treatment alone. However, CO_2 _elimination improved, suggesting better gas exchange with increased septation.

### Exercise swim testing

No difference in time to fatigue was noted on forced exercise swim testing for any group of rats (Saline/Control 45 ± 2, CSO/RA 45 ± 3, Dex, 45 ± 2, Dex + RA 39 ± 2 minutes, n = 6–8 per group). This suggests that, even in Dex-treated animals that demonstrated compromised pulmonary function by other measures, exercise tolerance was not affected by hormonal treatments.

### Extended study

Since Massaro and Massaro have previously demonstrated that RA promotes septation after the period of normal alveolarization [11] and our data showed that early RA effects were lost two weeks after stopping treatment, we next sought to determine whether the effects of later administration of RA were also reversed. Rat pups were normalized to 10 pups per litter and treated with either Dex or saline from days 1–14 followed by either CSO or RA from days 24–36 (12 days of treatment). Animals were studied at either day 37 (at the end of treatments) or day 52 (2 weeks after stopping treatment).

### Weight gain

Birth weights were the same for all animals (6.9 ± 0.1, n = 40). In untreated animals, growth velocities were similar until d24 after which males grew faster than females such that by d36 females weighed approximately 10% less than males (data not shown). Dex treatment affected both males and females equally with 8–9% lower weight at d14 (p < 0.01, n = 8 per group) and a 6–8% lower weight at d36 as compared to sex-matched controls (p = 0.09, n = 8 per group). As with the earlier study, RA treatment alone had no effect on weight (data not shown).

### Morphology

**Figure 5:**
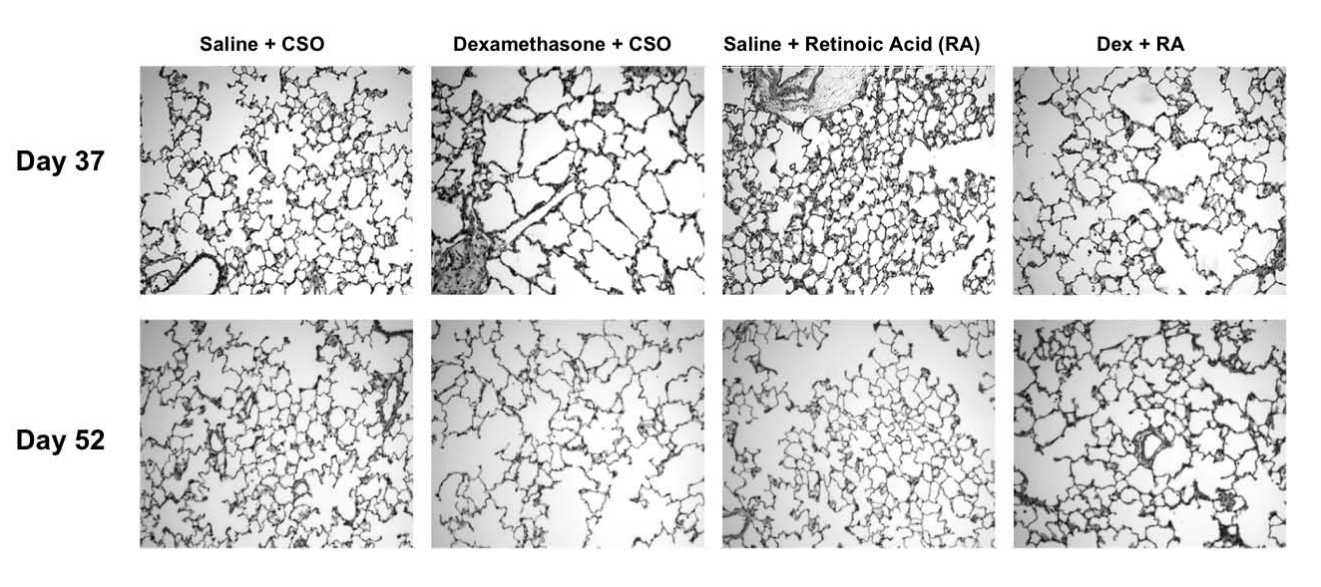
Effect of delayed RA treatment on alveolarization: Animals were treated with either Dex or saline from d1-14 followed by either CSO or RA from d24-36. A simplified distal architecture was seen in Dex-treated animals at both d37 (top) and d52 (bottom). RA-treated pups had smaller more numerous alveoli at d37 with changes no longer seen at d52. Early Dex + Later RA treatment resulted in restored secondary septation that was similar to that of saline controls at d37, and this was sustained to d52. (all images 100× magnification)

Alterations of lung architecture were similar to those seen with the Day 15/30 protocol (Fig. 5). At d37, Early Dex + Later CSO-treated animals had simplified distal air spaces compared to Early Saline + Later CSO controls. Early Saline + Later RA-treated pups, on the other hand, had smaller, more numerous alveoli than Early Saline + Later CSO controls. Normal architecture was restored in Early Dex + Later RA-exposed rats (Figure 5). Changes seen in the Early Dex + Later CSO group persisted at d52 while animals exposed to Early Dex + Later RA continued to have architecture similar to that of controls. Interestingly, at d52, Early Saline + Later RA-treated rats had lung histology that appeared similar to that of control animals (Figure 5), again demonstrating a loss of the effects of RA alone two weeks after treatment was stopped.

### Radial alveolar counts

RAC were used to quantify the changes in alveolarization in the extended study. In concordance with histological appearance, RAC were significantly lower in Early Dex + Later CSO-treated and significantly higher in Early Saline + Later RA-treated animals at d37 (Table 6). Rats treated with both hormones had RAC no different from controls. At d52, RAC remained significantly lower in Early Dex + Later CSO-treated animals compared to Early Saline + Later CSO controls, but both Early Saline + Later RA- and Early Dex + Later RA-treated animals were similar to controls thereby confirming the reversal of RA alone effects two weeks after stopping treatment (Table 6). The distribution of males and females in these studies was equal and no differences were noted between them with respect to the histology or RAC (data not shown).

**Table 6:** Radial alveolar counts (RAC) at d37 and d52: RAC were significantly lower in Early Dex + Later CSO-treated and higher in Early Saline + Later RA-treated pups while Early Dex + Later RA animals were similar to controls. At d52, while RAC continued to be significantly lower in Early Dex + Later CSO-treated pups, Early Saline + Later RA effects were lost two weeks after stopping RA treatment. Values are expressed mean ± SE. *p < 0.01 vs. d37 Early Saline + Later CSO-treated animals; †p < 0.01 vs. Early Dex + Late CSO; ¶p < 0.01 vs. Early Saline + late RA. CSO: Cottonseed oil.

**Treatment Group**	**Day 37**	**Day 52**
Control (n = 4)	9.1 ± 0.2	9.5 ± 0.2
Early Saline + Late CSO (n = 4)	9.3 ± 0.1	9.4 ± 0.1
Early Saline + Late RA (n = 4)	10.6 ± 0.1*	9.7 ± 0.1¶
Early Dex + Late CSO (n = 4)	7.7 ± 0.2*	7.8 ± 0.1*
Early Dex + Late RA (n = 4)	8.9 ± 0.3†	9.2 ± 0.1†

## Discussion

In the present study, we confirm hormonally mediated changes in architecture during postnatal lung development in the rat. Respiratory acidosis, the most significant functional abnormality, was noted on d15 in both RA alone-and Dex alone-treated rat pups and was resolved in Dex + RA-treated animals. However, Dex + RA failed to resolve the increased tachypnea, MV, and TV seen in Dex alone-treated rats. Massaro and Massaro have previously shown that Dex + RA treatment results in an increased body mass-specific surface area available for gas exchange compared to rats treated with Dex alone [10]. In the face of persistently larger lung volumes and equivalent body weight in both Dex-and Dex + RA-treated animals, the improved CO_2 _elimination in Dex + RA-treated animals is likely the effect of improved secondary septation and a larger surface area for gas exchange.

Interestingly, the increase in RAC on d15 in rats treated with RA alone was associated with hypercarbia, lower PaO_2 _and acidosis, but without any effect on other pulmonary function parameters studied. The reason for this abnormality in ABG is unclear, but suggests a defect in gas exchange. It is unlikely that this abnormality is due to the increased number of alveoli as it has previously been shown that RA treatment alone does not increase surface area [10]. However, ABG were normal in Dex + RA animals at d15, as well as in RA alone-treated pups by d30 when the RA alone-stimulated changes in distal lung structure had also resolved. Indeed, while the effects of Dex alone and concomitant RA administration were sustained for at least 15 days after stopping the treatments, the effects of RA alone from either d4 to d14 or d24 to d36 were reversed two weeks after stopping RA.

Alveolar development, the last phase of lung development, occurs either pre- or postnatally depending on the species. In the human, alveolarization begins in utero at about 36 weeks of gestation and continues postnatally, whereas in the rodent, secondary septation is an entirely postnatal event. Alveolarization appears to correlate inversely with changes in serum corticosteroid concentrations. It is likely that the normal timing of alveolar development reflects decreased corticosteroid levels leading to an increase in DNA synthesis and septation. For example, in the rat, corticosterone concentrations drop to a nadir between postnatal days 6 and 12, the time of maximum alveolar formation [8]. Conversely, administration of corticosteroid during this critical period results in an inhibition of alveolarization [9]. Indeed, exposure of fetal rhesus macaques to triamcinolone during the pseudoglandular and saccular phases of lung development results in an inhibition of septation [16]. The mechanisms of Dex-induced inhibition of alveolarization are likely multifactorial, including inhibition of DNA synthesis, differential regulation of matrix components, and changes in gene expression in the lung [17]. In addition, corticosteroids cause a growth retardation that is in itself associated with a slowing of alveolar growth [18].

Since the structural effects of Dex administration during the time of secondary septation are sustained to adulthood, the concept of a "critical period" of alveolar development was proposed. However, several lines of evidence support the notion that alveolar growth occurs throughout life and can be manipulated past the immediate newborn period. For example, starvation-induced decreases in alveolar formation are reversed upon refeeding [18]. In addition, treatment of rats previously exposed to Dex from d3 to d15 with RA from d24 to d36 results in a restitution of Dex-induced simplification of the distal lung [11]. Most promising for clinical practice, however, is the ability of RA to stimulate alveolar formation in adult rats after emphysema was induced by intratracheal instillation of elastase [19]. On the other hand, in the present study, the effects of RA alone were not sustained after discontinuation of treatment.

The mechanisms of RA effects on alveolarization are likely via changes in the expression of epithelial (e.g. VEGF) and mesenchymal (e.g. PDGF and TGFβ) growth factors critical for cell proliferation and differentiation, angiogenesis, and matrix deposition during lung development [20,21]. In addition, the regulation of free all-*trans*-RA by RA-binding proteins and interactions with RA receptors (RAR and RXR) contribute to appropriate lung development. For example, RAR-α promotes epithelial cell differentiation during the progression from pseudoglandular to canalicular stages of lung development [20]. In addition, RAR-α also promotes alveolar formation after the perinatal period [22]. Meanwhile, the expression of RAR-β increases towards the end of the saccular stage corresponding to an induction of both type 1 and type 2 epithelial cells [20]. However, RAR-β knockout mice exhibit premature septation and RAR-β agonist treatment of neonatal rats results in impaired septation, thereby identifying RAR-β as an inhibitor of alveolar formation [23]. On the other hand, impaired distal airspace formation during postnatal lung development has also been reported in RAR-β knockout mice [24]. Finally, targeted deletion of RAR-γ in mice is associated with a decrease in alveolar number, suggesting the importance of this receptor in the development of normal alveoli [25]. In our study, while Dex + RA treatment prevented some structural effects seen with Dex alone, effects due to RA alone were reversible. This suggests that mechanisms are in place to normalize alveolar structure, but these mechanism(s), in particular those leading to the reversal of RA effects, are currently unknown.

To date, there has been a paucity of literature on the effects of hormone-induced structural changes on pulmonary function. Srinivasan et al. examined several lung variables including RR, MV, and TV in sedated animals at 30 to 39 days of life and noted no differences in any treatment group [12]. While our findings of increased lung volumes in PV curves of Dex-treated animals mirror those of Srinivasan et al., our study, performed at day 13 in non-sedated animals, showed tachypnea and increased TV and MV in Dex-treated animals. At d15 and d30 in Dex-treated animals, blood gases obtained in anesthesized, but spontaneously breathing animals revealed a respiratory acidosis despite an increased MV confirming significant compromise in pulmonary function. It is likely that the respiratory acidosis and consequent tachypnea are due to increased dead space in affected animals. Interestingly, while lung volume differences were not resolved, RAC and blood gases were normalized with Dex + RA treatment, suggesting that correction of blood gas abnormalities likely resulted from an increase in the surface area available for CO_2 _elimination rather than full correction of lung volumes. Further, the mechanisms leading to the respiratory acidosis and lower PaO_2 _seen with RA alone treatment at d15 are unknown. However, these changes were not evident in Dex + RA pups at d15 or at d30 when the RA alone-induced changes had reversed, suggesting that the altered architecture seen in RA alone-treated pups may have contributed to the blood gas abnormalities.

Varying degrees of exercise intolerance have been described in patients with emphysema [26] and BPD [27]. In our study, no differences in exercise tolerance were found with any treatment. This suggests that factors other than altered alveolar structure, such as fibrosis and restrictive/obstructive lung disease, may play a significant role in the diminished exercise tolerance observed in affected patients.

Lastly, starvation and decreased body weight are associated with decreased alveolarization [18]. However, in our studies, while weight was decreased in both Dex- and Dex + RA-treated animals, Dex + RA animals had a restitution of secondary septation, suggesting that distal lung structure can be manipulated independent of body weight. This is important in preterm neonates that have significantly compromised nutrition and poor weight gain in addition to an arrest of alveolarization.

## Conclusion

In summary, hormonal treatment of rat pups results in altered lung architecture. This is associated with significant structure-function disturbances where Dex-induced decreases in alveolarization are associated with increased lung volumes, CO_2 _retention, acidosis, and tachypnea. Our findings showing that the effects of RA on generation of alveoli and on gas exchange appear to be time-limited and reversible may be relevant for the use of RA in treatment of diseases such as BPD or emphysema.

## Competing interests

The author(s) declare that they have no competing interests.

## Authors' contributions

SJG was responsible for injecting and harvesting all animals, measuring radial alveolar counts, performing all functional and statistical analyses, and drafting the manuscript. HZ was responsible for generating all the blood gas and volume of displacement data at day 15, interpretation and revision of the manuscript. JPF assisted in animal harvesting and injections as well as conducting functional studies. RIG and MHG assisted in obtaining the pressure volume measurements. AJG assisted in the analysis of PV curves. RCS conceived the study, participated in its design and coordination, and helped draft and revise the manuscript. All authors read and approved the final manuscript.
